# Supplementary material for: Hundreds of grocery outlets needed across the United States to achieve walkable cities
Source: Nat Commun. 2025 Jul 1;16:6051. doi: 10.1038/s41467-025-61454-1 (PMC12214625; doi:10.1038/s41467-025-61454-1)
Supplement: Supplementary file 1 — Supplementary Information [file 41467_2025_61454_MOESM1_ESM.pdf]

Supplementary Table: City Rankings and Supermarket Additions

Supplementary Table 1. City-level equity-penalized distance (EDE) rankings. Cities are sorted by EDE, which reflects the average walking distance to the nearest supermarket weighted by population and penalized for inequality. Additional columns show the number of additional supermarkets placed at each city under different optimization strategies (maximizing coverage within 5, 10, or 15 minutes).

				Additional Supermarkets			
**City, State**	**Rank**	**EDE (m)**	**Population**	**Avg**	**15 min**	**10 min**	**5 min**
Union City, NJ	1	579.3	68,186	0	0	0	3
Santa Monica, CA	2	764.2	92,812	0	0	0	22
New York, NY	3	800.2	8,784,592	0	0	2	440
Jersey City, NJ	4	832.7	291,585	0	0	1	31
Cambridge, MA	5	851.2	117,858	0	0	1	21
Inglewood, CA	6	948.9	106,817	0	0	2	44
San Francisco, CA	7	968.5	871,136	0	0	9	127
Redondo Beach, CA	8	994.1	71,344	0	0	3	27
Berkeley, CA	9	995.2	123,485	0	0	2	30
Hawthorne, CA	10	1005.8	87,911	0	0	3	31
Somerville, MA	11	1011.7	80,995	0	0	2	17
Miami, FL	12	1039.6	441,228	0	0	12	120
Burbank, CA	13	1041.4	104,508	0	0	3	52
South Gate, CA	14	1062.3	91,627	0	0	4	34
Philadelphia, PA	15	1063.9	1,593,147	0	0	27	329
Santa Clara, CA	16	1064.8	126,522	0	0	7	–
Washington, DC	17	1073.5	684,900	0	0	15	182
Providence, RI	18	1080.2	189,588	0	0	6	66
Long Beach, CA	19	1089.5	464,262	0	0	15	164
Chicago, IL	20	1093.2	2,733,239	0	0	43	553
Hialeah, FL	21	1094.8	222,413	0	0	7	–
Newark, NJ	22	1098.7	310,849	0	0	5	79
Paterson, NJ	23	1103.9	159,216	0	0	2	26
Lakewood, CA	24	1109.3	82,198	0	0	7	–
Evanston, IL	25	1111.0	77,617	0	0	4	29
Seattle, WA	26	1115.5	726,482	0	0	27	311
Mount Vernon, NY	27	1116.8	73,645	0	0	2	18
Alexandria, VA	28	1129.8	157,507	0	0	10	64
Passaic, NJ	29	1139.6	70,297	0	0	2	12
Cicero, IL	30	1152.9	85,026	0	0	2	14
Whittier, CA	31	1155.0	85,826	0	0	8	–
New Rochelle, NY	32	1167.2	77,661	0	0	6	–
Pasadena, CA	33	1171.7	135,215	0	0	10	–
Lowell, MA	34	1174.9	112,487	0	0	8	–
Santa Ana, CA	35	1182.1	309,348	0	0	15	–
Sunnyvale, CA	36	1190.5	154,895	0	0	14	–
Bellflower, CA	37	1190.9	79,056	0	0	6	–
Lawrence, MA	38	1191.8	88,522	0	0	4	28
Baldwin Park, CA	39	1193.9	71,795	0	0	6	–
Huntington Beach, CA	40	1196.2	196,446	0	0	20	–
Lynwood, CA	41	1210.2	67,083	0	1	3	25
Daly City, CA	42	1222.8	104,020	0	1	7	–
Yonkers, NY	43	1224.4	210,151	0	1	13	105
Santa Barbara, CA	44	1234.1	83,661	0	1	10	–
Pawtucket, RI	45	1234.5	74,767	0	1	5	36
Hartford, CT	46	1241.3	119,089	0	1	8	–
Elizabeth, NJ	47	1248.1	136,788	0	1	5	28

City, State	Rank	EDE (m)	Population	Additional Supermarkets Avg	15 min	10 min	5 min
Syracuse, NY	48	1248.3	145,796	0	1	13	–
San Mateo, CA	49	1249.1	104,267	0	1	8	–
Torrance, CA	50	1252.3	146,799	0	1	14	–
Buena Park, CA	51	1258.8	83,271	0	1	9	–
Downey, CA	52	1259.8	114,624	0	1	12	–
El Cajon, CA	53	1298.0	104,535	0	1	14	–
Buffalo, NY	54	1303.8	274,210	0	2	17	152
Erie, PA	55	1306.6	92,128	0	1	10	–
Los Angeles, CA	56	1308.9	3,849,235	0	8	186	2216
Boston, MA	57	1309.6	670,755	0	2	21	161
Fullerton, CA	58	1314.6	141,320	0	2	22	–
Glendale, CA	59	1315.8	192,354	0	2	12	–
Tempe, AZ	60	1321.4	176,910	0	2	27	–
Costa Mesa, CA	61	1336.9	109,236	0	2	12	–
Alhambra, CA	62	1337.4	82,703	0	1	5	–
Fort Lauderdale, FL	63	1357.1	181,895	0	3	28	–
St. Louis, MO	64	1372.9	298,399	0	4	28	–
Rochester, NY	65	1373.9	208,334	0	3	19	–
Trenton, NJ	66	1382.7	90,014	0	1	5	31
Mountain View, CA	67	1388.1	81,213	0	1	8	–
Beaverton, OR	68	1391.0	95,542	0	3	17	–
Garden Grove, CA	69	1391.3	171,455	0	3	20	–
Oakland, CA	70	1396.6	432,343	0	4	28	212
Alameda, CA	71	1409.1	76,367	0	2	6	32
Yakima, WA	72	1419.4	87,551	0	3	19	–
Baltimore, MD	73	1421.2	577,766	0	6	40	270
Allentown, PA	74	1424.1	123,746	0	1	8	54
Ontario, CA	75	1436.9	172,041	0	4	25	–
Bellingham, WA	76	1439.1	80,221	0	3	18	–
Miami Beach, FL	77	1442.3	80,471	0	1	5	26
Portland, OR	78	1454.9	634,209	0	9	66	–
Racine, WI	79	1455.9	76,989	0	2	9	69
Reading, PA	80	1462.7	93,921	0	1	5	25
Pomona, CA	81	1463.9	149,957	0	4	19	–
Wilmington, DE	82	1465.8	70,141	0	1	4	29
Everett, WA	83	1471.1	103,436	0	3	16	–
Albany, NY	84	1472.5	95,045	0	2	7	–
Hollywood, FL	85	1475.1	152,077	0	5	25	–
Ventura, CA	86	1478.8	107,880	0	3	21	–
Anaheim, CA	87	1480.7	343,296	0	8	44	–
Norwalk, CA	88	1503.0	102,487	0	2	9	–
Bridgeport, CT	89	1514.8	147,033	0	4	12	84
Brockton, MA	90	1517.8	100,074	0	4	18	–
Arlington Heights, IL	91	1522.8	75,066	0	5	17	–
Tustin, CA	92	1525.6	79,005	0	3	12	–
Boulder, CO	93	1531.5	105,128	0	3	15	–
Santa Maria, CA	94	1533.3	108,035	0	3	10	–
Cleveland, OH	95	1539.1	363,467	0	8	44	–
Compton, CA	96	1539.8	95,422	0	1	5	45
Turlock, CA	97	1542.2	71,557	0	2	12	–
Upland, CA	98	1544.4	77,572	0	3	17	–
San Jose, CA	99	1555.4	993,779	0	19	121	–
Milwaukee, WI	100	1569.0	564,921	0	13	56	438
Glendale, AZ	101	1569.5	241,320	0	10	50	–
Corona, CA	102	1574.8	151,544	0	6	32	–
New Bedford, MA	103	1575.1	97,844	0	1	6	72
Norfolk, VA	104	1576.9	231,618	0	8	44	–
Spokane, WA	105	1582.3	213,797	0	10	42	–

City, State	Rank	EDE (m)	Population	Additional Supermarkets Avg	15 min	10 min	5 min
Mission Viejo, CA	106	1582.5	92,490	0	7	27	–
Redwood City, CA	107	1586.9	82,797	0	2	8	–
Milpitas, CA	108	1587.9	79,444	0	2	14	–
Oxnard, CA	109	1600.5	200,281	0	3	22	–
Denver, CO	110	1607.5	705,515	0	14	72	–
San Leandro, CA	111	1608.5	90,139	0	2	8	–
Portland, ME	112	1613.2	63,481	0	3	11	–
Honolulu, HI	113	1616.4	341,854	0	6	26	148
Bellevue, WA	114	1629.8	136,046	0	9	36	–
Vista, CA	115	1632.6	96,566	0	7	35	–
Largo, FL	116	1633.8	78,838	0	6	32	–
Iowa City, IA	117	1644.8	67,677	0	6	24	–
Miami Gardens, FL	118	1647.8	110,828	0	6	21	–
Vancouver, WA	119	1649.4	172,975	0	10	42	–
Salinas, CA	120	1659.3	161,947	0	4	16	–
Irvine, CA	121	1659.7	302,364	0	9	45	–
Centennial, CO	122	1660.1	100,783	0	8	39	–
Allen, TX	123	1661.3	101,915	0	7	31	–
Orem, UT	124	1665.5	94,784	0	5	19	–
Richardson, TX	125	1674.4	116,636	0	6	30	–
Riverside, CA	126	1675.8	299,063	0	13	65	–
Union City, CA	127	1678.2	68,266	0	3	11	–
Gresham, OR	128	1679.3	107,907	0	6	29	–
Newport Beach, CA	129	1685.4	81,967	0	8	24	–
Tacoma, WA	130	1686.8	208,299	0	7	32	–
Dearborn, MI	131	1689.7	107,471	0	4	13	–
Sacramento, CA	132	1709.0	517,871	0	20	88	–
New Britain, CT	133	1709.2	71,157	0	4	13	–
Schenectady, NY	134	1712.9	65,747	0	2	9	–
Mesa, AZ	135	1713.1	484,305	0	29	146	–
Pleasanton, CA	136	1714.3	71,513	0	5	27	–
Davenport, IA	137	1717.3	89,289	0	8	31	–
Grand Rapids, MI	138	1728.3	190,044	0	9	40	–
Pembroke Pines, FL	139	1732.7	157,334	1	16	52	–
Orange, CA	140	1733.5	135,952	0	7	28	–
Pompano Beach, FL	141	1740.4	110,941	0	8	23	–
Bethlehem, PA	142	1743.4	71,984	0	4	12	–
San Diego, CA	143	1745.3	1,347,374	0	51	201	–
Hemet, CA	144	1747.9	86,581	0	8	36	–
Carson, CA	145	1748.7	94,353	0	3	13	–
Boise City, ID	146	1751.8	214,257	0	21	88	–
Hammond, IN	147	1754.1	74,652	0	5	14	–
Des Moines, IA	148	1754.8	197,338	0	13	55	–
Fontana, CA	149	1757.5	202,227	0	11	46	–
Kenner, LA	150	1758.1	65,724	0	4	13	–
Boca Raton, FL	151	1758.5	96,111	0	11	36	–
Antioch, CA	152	1765.7	109,165	0	7	31	–
Ann Arbor, MI	153	1767.9	115,073	0	6	21	–
Westminster, CA	154	1769.2	90,842	0	3	14	–
Fremont, CA	155	1778.7	223,694	0	10	38	–
San Bernardino, CA	156	1779.1	213,770	0	9	42	–
Redlands, CA	157	1780.9	67,242	0	4	19	–
Thousand Oaks, CA	158	1781.8	109,670	0	16	–	–
Cary, NC	159	1784.8	151,115	0	27	–	–
Kent, WA	160	1789.6	98,383	0	9	36	–
Plano, TX	161	1789.9	278,185	0	18	83	–
Hayward, CA	162	1794.8	159,235	0	5	23	–
Clearwater, FL	163	1797.1	108,566	0	8	34	–

City, State	Rank	EDE (m)	Population	Additional Supermarkets Avg	15 min	10 min	5 min
Rancho Cucamonga, CA	164	1800.6	169,450	0	12	52	–
Federal Way, WA	165	1809.0	95,059	0	6	28	–
Lynchburg, VA	166	1811.9	60,740	0	9	32	–
Pasadena, TX	167	1813.5	146,356	0	11	41	–
Deerfield Beach, FL	168	1819.1	86,410	0	7	24	–
Chandler, AZ	169	1820.5	267,830	0	19	82	–
Hillsboro, OR	170	1821.6	102,863	0	5	21	–
Chula Vista, CA	171	1823.7	267,847	0	11	43	–
Roanoke, VA	172	1824.7	88,934	0	11	45	–
Cranston, RI	173	1824.7	76,714	0	6	23	–
Eugene, OR	174	1827.1	167,768	0	11	45	–
West Covina, CA	175	1831.4	107,706	0	8	27	–
Lake Forest, CA	176	1834.1	85,226	0	4	23	–
Worcester, MA	177	1835.8	194,968	0	9	34	–
Renton, WA	178	1836.3	101,133	0	9	30	–
Plantation, FL	179	1837.9	90,340	0	11	39	–
Concord, CA	180	1839.2	123,107	0	7	27	–
Westminster, CO	181	1839.9	111,754	0	10	40	–
Asheville, NC	182	1845.0	82,804	0	18	–	–
Pittsburgh, PA	183	1848.2	295,407	0	13	42	–
Arlington, TX	184	1850.3	381,169	0	27	108	–
Kennewick, WA	185	1852.7	75,061	0	9	–	–
Napa, CA	186	1857.3	77,597	0	4	20	–
Bloomington, IN	187	1872.5	74,634	0	8	28	–
Sandy Springs, GA	188	1873.7	96,024	0	13	47	–
Gainesville, FL	189	1873.8	131,561	0	12	44	–
Medford, OR	190	1873.8	80,861	0	8	–	–
Minneapolis, MN	191	1876.3	426,006	0	9	34	203
Salt Lake City, UT	192	1876.9	192,419	0	8	28	–
Hampton, VA	193	1879.3	129,399	0	18	–	–
Lewisville, TX	194	1884.9	108,170	0	9	41	–
Rialto, CA	195	1886.1	101,915	0	4	17	–
Westland, MI	196	1889.2	80,668	0	7	28	–
Salem, OR	197	1895.6	166,113	0	17	–	–
Lauderhill, FL	198	1896.4	74,339	0	6	14	–
Mesquite, TX	199	1897.0	143,400	0	13	48	–
Palatine, IL	200	1903.9	65,935	0	6	26	–
Richmond, CA	201	1905.1	112,469	0	7	25	–
Simi Valley, CA	202	1905.6	117,397	0	8	34	–
Evansville, IN	203	1909.0	105,228	0	11	38	–
Fresno, CA	204	1910.0	526,741	0	28	114	–
Ogden, UT	205	1917.3	83,221	0	8	24	–
Visalia, CA	206	1919.8	137,491	0	9	42	–
Fall River, MA	207	1921.6	90,403	0	3	12	–
Manteca, CA	208	1922.9	75,369	0	6	22	–
Houston, TX	209	1927.0	2,215,641	0	112	469	–
Springfield, MA	210	1934.3	148,060	0	7	27	–
Lakewood, CO	211	1937.3	152,267	0	12	44	–
San Marcos, CA	212	1937.8	87,032	0	9	–	–
Longmont, CO	213	1939.6	94,633	0	7	25	–
Champaign, IL	214	1940.1	84,999	0	7	21	–
Elgin, IL	215	1944.4	107,031	0	10	70	–
Chico, CA	216	1945.6	95,131	0	7	28	–
Carrollton, TX	217	1945.6	130,380	0	11	35	–
Sioux City, IA	218	1947.1	73,047	0	6	19	–
Durham, NC	219	1951.2	243,313	0	37	–	–
West Palm Beach, FL	220	1953.2	113,725	0	9	37	–
Missoula, MT	221	1955.7	62,101	0	10	31	–

City, State	Rank	EDE (m)	Population	Additional Supermarkets Avg	15 min	10 min	5 min
Citrus Heights, CA	222	1957.3	86,347	0	6	26	–
Canton, OH	223	1958.1	68,119	0	6	18	–
Sparks, NV	224	1962.4	100,003	0	8	35	–
Orlando, FL	225	1966.2	292,032	0	24	154	–
Gastonia, NC	226	1967.3	64,689	0	25	–	–
Farmington Hills, MI	227	1982.3	69,330	0	12	35	–
Lake Charles, LA	228	1985.5	74,947	0	13	51	–
Charleston, SC	229	1986.1	126,534	0	40	–	–
St. Petersburg, FL	230	1989.7	253,993	0	13	52	–
Clifton, NJ	231	1992.7	88,716	0	4	14	–
Charlotte, NC	232	1994.4	804,437	0	126	–	–
Raleigh, NC	233	1998.2	430,197	0	56	–	–
Bolingbrook, IL	234	2002.5	69,312	0	8	29	–
Las Vegas, NV	235	2004.5	623,239	0	37	147	–
Detroit, MI	236	2010.0	625,092	0	29	98	–
Oceanside, CA	237	2016.6	169,037	0	19	54	–
Greenville, NC	238	2017.3	74,560	0	17	–	–
Davie, FL	239	2020.5	98,467	0	25	–	–
Dayton, OH	240	2024.8	130,685	0	12	36	–
Toledo, OH	241	2031.6	255,596	0	20	64	–
Modesto, CA	242	2036.6	213,408	0	10	39	–
Omaha, NE	243	2045.3	409,421	0	30	102	–
Decatur, IL	244	2047.3	60,573	0	13	57	–
Schaumburg, IL	245	2049.5	77,243	0	7	32	–
Kenosha, WI	246	2052.5	93,324	0	7	24	–
El Monte, CA	247	2054.2	108,897	0	4	12	–
Scranton, PA	248	2055.1	72,026	0	4	13	–
Merced, CA	249	2059.9	84,389	0	7	26	–
Portsmouth, VA	250	2060.8	94,416	0	10	32	–
Cedar Rapids, IA	251	2061.3	116,963	0	15	80	–
Lafayette, LA	252	2064.1	108,594	0	20	58	–
Escondido, CA	253	2070.3	143,784	0	17	–	–
Garland, TX	254	2071.7	238,299	0	13	58	–
Wilmington, NC	255	2073.5	102,584	0	19	–	–
Santa Clarita, CA	256	2079.2	172,029	0	15	61	–
Sunrise, FL	257	2079.7	96,314	0	11	–	–
Fayetteville, NC	258	2082.4	179,552	0	45	–	–
Appleton, WI	259	2087.3	70,697	0	7	20	–
Columbus, OH	260	2090.0	868,417	0	81	270	–
Lynn, MA	261	2090.8	100,574	0	3	8	–
Newton, MA	262	2095.8	85,382	0	6	19	–
Tampa, FL	263	2096.1	375,087	0	28	101	–
Lexington, KY	264	2108.3	300,774	0	30	140	–
Southfield, MI	265	2113.1	67,813	0	10	37	–
Livonia, MI	266	2114.1	84,614	0	14	61	–
Wyoming, MI	267	2115.4	70,740	0	10	36	–
Richmond, VA	268	2124.0	216,832	0	15	46	–
Parma, OH	269	2125.6	76,263	0	6	21	–
Rock Hill, SC	270	2127.8	62,130	0	17	–	–
Warren, MI	271	2131.0	132,813	0	13	39	–
Boynton Beach, FL	272	2135.1	79,303	0	8	23	–
Vacaville, CA	273	2137.5	98,718	0	9	–	–
Thornton, CO	274	2139.1	136,466	0	15	54	–
Dallas, TX	275	2139.4	1,269,024	0	63	256	–
Green Bay, WI	276	2140.6	95,923	0	10	37	–
Murfreesboro, TN	277	2146.6	133,654	0	39	–	–
Elk Grove, CA	278	2148.0	167,695	0	11	38	–
Moreno Valley, CA	279	2148.9	199,624	0	15	54	–

City, State	Rank	EDE (m)	Population	Additional Supermarkets Avg	15 min	10 min	5 min
McKinney, TX	280	2156.2	181,632	0	23	79	–
New Haven, CT	281	2160.7	131,263	0	4	13	–
Akron, OH	282	2161.7	177,615	0	17	52	–
Mobile, AL	283	2163.6	163,412	0	37	–	–
Colorado Springs, CO	284	2169.1	441,729	0	57	257	–
Sugar Land, TX	285	2176.5	78,571	0	18	–	–
Brooklyn Park, MN	286	2177.4	78,523	0	9	29	–
Carlsbad, CA	287	2184.4	109,493	0	15	–	–
Overland Park, KS	288	2193.5	181,921	0	22	79	–
Johns Creek, GA	289	2194.2	74,060	0	24	–	–
Missouri City, TX	290	2194.7	71,184	0	14	–	–
Rockford, IL	291	2194.8	132,663	0	15	46	–
Beaumont, TX	292	2207.6	102,845	0	21	–	–
West Valley City, UT	293	2213.1	137,151	0	11	42	–
Arvada, CO	294	2214.2	113,007	0	14	51	–
Newport News, VA	295	2214.4	177,661	0	19	62	–
Lawrence, KS	296	2214.9	88,713	0	9	32	–
Tracy, CA	297	2215.7	89,203	0	9	29	–
Santa Fe, NM	298	2220.7	65,422	0	15	–	–
Chino, CA	299	2221.6	88,988	0	7	35	–
Knoxville, TN	300	2221.7	165,716	0	38	–	–
Bend, OR	301	2232.8	86,052	0	14	–	–
South Bend, IN	302	2233.8	96,510	0	10	32	–
Flint, MI	303	2234.8	73,988	0	11	28	–
Irving, TX	304	2236.2	249,508	0	16	54	–
Bryan, TX	305	2240.5	72,845	0	20	–	–
Palmdale, CA	306	2241.1	163,712	0	26	–	–
Murrieta, CA	307	2243.9	103,793	0	13	40	–
Waterbury, CT	308	2246.2	105,829	0	10	31	–
Loveland, CO	309	2249.5	70,084	0	12	–	–
Gilbert, AZ	310	2252.2	258,535	0	30	118	–
Lafayette, IN	311	2253.2	63,860	0	8	25	–
Greeley, CO	312	2253.6	101,471	0	12	45	–
Springfield, MO	313	2257.2	156,097	0	26	88	–
Manchester, NH	314	2261.2	104,461	0	10	35	–
Greensboro, NC	315	2263.2	275,555	0	64	–	–
Temecula, CA	316	2267.4	105,794	0	15	–	–
Quincy, MA	317	2270.8	99,023	0	6	14	–
Naperville, IL	318	2273.1	143,475	0	15	58	–
Fort Collins, CO	319	2277.1	157,803	0	17	65	–
Avondale, AZ	320	2277.9	86,924	0	13	36	–
Phoenix, AZ	321	2281.7	1,553,053	0	80	358	–
Cheyenne, WY	322	2284.8	60,472	0	7	21	–
Chattanooga, TN	323	2291.7	144,809	1	40	–	–
Roswell, GA	324	2294.3	79,424	1	28	–	–
Livermore, CA	325	2320.1	82,877	1	13	–	–
Pearland, TX	326	2325.9	116,526	1	22	–	–
Stockton, CA	327	2328.8	315,003	1	16	60	–
North Las Vegas, NV	328	2330.7	258,748	1	20	64	–
San Ramon, CA	329	2333.3	77,129	1	8	26	–
Santa Rosa, CA	330	2336.2	158,264	1	11	47	–
Atlanta, GA	331	2337.6	460,547	1	33	123	–
Peoria, IL	332	2339.8	100,459	1	16	67	–
Indio, CA	333	2342.7	82,254	1	14	38	–
Peoria, AZ	334	2349.6	180,203	1	40	–	–
Tyler, TX	335	2353.4	91,719	1	25	–	–
Pueblo, CO	336	2355.1	105,150	1	12	32	–
Little Rock, AR	337	2355.7	173,674	1	54	–	–

City, State	Rank	EDE (m)	Population	Additional Supermarkets Avg	15 min	10 min	5 min
Rochester, MN	338	2355.9	99,740	1	21	–	–
Fayetteville, AR	339	2356.7	78,871	1	27	–	–
Warner Robins, GA	340	2361.9	70,494	1	29	–	–
Mount Pleasant, SC	341	2365.5	71,880	1	–	–	–
Folsom, CA	342	2371.2	76,978	1	16	–	–
Norwalk, CT	343	2376.4	81,524	1	8	26	–
Springdale, AR	344	2377.4	70,967	1	18	–	–
Camden, NJ	345	2388.6	71,190	1	3	7	36
Independence, MO	346	2390.7	106,725	1	24	76	–
Meridian, ID	347	2401.4	98,984	1	13	44	–
Scottsdale, AZ	348	2401.8	205,084	1	62	–	–
Muncie, IN	349	2403.7	57,695	1	8	23	–
Tallahassee, FL	350	2407.8	181,092	1	42	–	–
Deltona, FL	351	2408.8	82,602	1	18	–	–
Waukegan, IL	352	2409.4	85,092	1	8	26	–
Coral Springs, FL	353	2429.5	133,606	1	–	–	–
Sioux Falls, SD	354	2433.5	172,902	1	23	100	–
Troy, MI	355	2440.9	76,482	1	14	–	–
Stamford, CT	356	2446.0	116,053	1	8	20	–
Rochester Hills, MI	357	2446.7	63,088	1	18	–	–
Birmingham, AL	358	2467.0	180,448	2	37	115	–
Youngstown, OH	359	2473.3	51,629	1	11	30	–
Melbourne, FL	360	2487.8	80,608	1	22	–	–
Cincinnati, OH	361	2487.8	298,967	1	27	83	–
Chino Hills, CA	362	2500.0	67,639	1	11	–	–
Columbia, SC	363	2503.9	121,572	1	31	–	–
Bakersfield, CA	364	2505.5	391,761	1	28	113	–
Madison, WI	365	2508.6	255,295	1	19	64	–
Macon, GA	366	2519.0	75,294	1	17	56	–
Sterling Heights, MI	367	2524.0	127,785	1	17	54	–
Spokane Valley, WA	368	2525.4	92,329	1	15	–	–
Aurora, IL	369	2528.4	171,214	1	17	59	–
Indianapolis, IN	370	2539.5	788,869	3	121	462	–
Fort Wayne, IN	371	2539.8	237,047	2	43	–	–
Vallejo, CA	372	2546.7	121,109	1	10	31	–
Louisville, KY	373	2557.1	567,257	3	89	337	–
Kalamazoo, MI	374	2563.1	67,303	1	8	24	–
Nampa, ID	375	2567.7	92,738	1	15	–	–
Nashville, TN	376	2585.7	588,971	5	156	–	–
Roseville, CA	377	2587.2	139,356	1	13	52	–
Frisco, TX	378	2594.9	187,075	1	27	–	–
Provo, UT	379	2595.6	110,239	1	8	25	–
Winston-Salem, NC	380	2610.8	202,683	2	89	–	–
Olathe, KS	381	2611.5	127,633	1	21	65	–
Clovis, CA	382	2617.8	110,618	1	8	30	–
Baytown, TX	383	2622.8	78,510	2	17	–	–
Chesapeake, VA	384	2624.5	207,651	3	–	–	–
Lansing, MI	385	2630.2	106,227	2	13	37	–
Lancaster, CA	386	2630.8	163,417	1	16	–	–
Albuquerque, NM	387	2639.0	549,255	3	48	156	–
San Antonio, TX	388	2641.9	1,381,080	7	181	884	–
Henderson, NV	389	2644.6	303,432	2	41	–	–
North Charleston, SC	390	2646.5	102,806	2	–	–	–
Burlington, VT	391	2646.5	42,520	1	3	8	–
Layton, UT	392	2650.0	72,740	2	12	–	–
Charleston, WV	393	2668.3	36,278	2	15	–	–
Yuma, AZ	394	2684.5	87,450	1	10	51	–
Memphis, TN	395	2692.6	585,130	3	77	270	–

City, State	Rank	EDE (m)	Population	Additional Supermarkets Avg	15 min	10 min	5 min
Lubbock, TX	396	2702.2	238,590	3	28	92	–
Danbury, CT	397	2702.3	68,197	1	13	45	–
League City, TX	398	2706.0	107,397	1	20	58	–
Jonesboro, AR	399	2711.0	55,667	2	22	–	–
St. Paul, MN	400	2725.5	306,600	1	14	39	–
El Paso, TX	401	2731.1	640,366	3	61	198	–
Corpus Christi, TX	402	2733.7	295,863	3	44	143	–
Lakeland, FL	403	2734.2	102,468	1	19	–	–
Springfield, IL	404	2736.7	101,948	2	28	–	–
Waterloo, IA	405	2747.1	57,317	2	10	31	–
Redding, CA	406	2749.5	71,725	2	41	–	–
Topeka, KS	407	2759.0	115,006	2	19	60	–
McAllen, TX	408	2775.0	131,993	2	15	53	–
High Point, NC	409	2775.6	97,657	2	31	–	–
Kansas City, MO	410	2785.6	453,839	3	64	209	–
Grand Prairie, TX	411	2788.0	188,187	2	27	–	–
Fairfield, CA	412	2793.0	112,955	1	14	–	–
Fargo, ND	413	2794.6	117,190	2	13	38	–
Mission, TX	414	2804.4	79,832	2	16	–	–
Longview, TX	415	2804.6	67,431	2	20	–	–
Warwick, RI	416	2807.4	73,689	2	14	50	–
Savannah, GA	417	2811.0	137,251	2	24	–	–
Montgomery, AL	418	2858.6	171,788	4	51	–	–
Baton Rouge, LA	419	2882.1	210,391	3	38	116	–
Broken Arrow, OK	420	2885.9	93,647	3	31	–	–
Sandy, UT	421	2889.2	90,722	1	12	45	–
College Station, TX	422	2896.3	113,469	2	16	–	–
Bloomington, MN	423	2922.5	81,089	1	14	37	–
Lee's Summit, MO	424	2928.4	87,205	3	28	–	–
Plymouth, MN	425	2955.2	64,676	2	18	–	–
Columbus, GA	426	2961.1	182,045	4	–	–	–
San Angelo, TX	427	2969.5	87,305	1	13	46	–
Hesperia, CA	428	2969.7	87,113	2	17	–	–
Auburn, WA	429	2970.6	79,338	2	11	31	–
Nashua, NH	430	2976.3	80,343	1	12	37	–
Las Cruces, NM	431	2988.1	103,326	2	21	–	–
Tulsa, OK	432	3022.7	380,249	3	45	163	–
Fort Smith, AR	433	3036.1	77,013	2	18	–	–
Round Rock, TX	434	3041.8	104,897	3	20	–	–
Hoover, AL	435	3056.6	76,966	6	37	–	–
Victorville, CA	436	3079.8	127,018	4	27	–	–
Killeen, TX	437	3098.4	139,105	2	19	77	–
Gary, IN	438	3098.6	62,903	2	11	34	–
Fishers, IN	439	3109.7	87,028	4	–	–	–
Menifee, CA	440	3113.0	90,654	2	22	–	–
Athens, GA	441	3139.5	95,851	2	52	–	–
Edmond, OK	442	3147.7	72,976	3	27	–	–
Reno, NV	443	3157.7	242,706	3	33	106	–
Kansas City, KS	444	3167.0	128,020	4	26	81	–
Concord, NC	445	3169.1	81,727	4	–	–	–
Billings, MT	446	3199.0	104,701	2	25	–	–
Wichita, KS	447	3204.4	360,255	10	71	–	–
Albany, GA	448	3233.7	57,411	3	24	–	–
Fort Worth, TX	449	3310.1	865,707	8	103	371	–
Suffolk, VA	450	3315.3	57,328	4	26	–	–
Laredo, TX	451	3340.9	242,729	4	26	78	–
Joliet, IL	452	3344.8	141,960	3	24	–	–
Aurora, CO	453	3344.9	374,055	1	30	138	–

City, State	Rank	EDE (m)	Population	Additional Supermarkets Avg	15 min	10 min	5 min
Augusta, GA	454	3346.5	156,034	9	84	–	–
Norman, OK	455	3373.0	104,271	2	19	58	–
O'Fallon, MO	456	3373.9	83,465	4	30	–	–
Clarksville, TN	457	3376.7	132,241	7	–	–	–
Apple Valley, CA	458	3378.7	62,164	4	25	–	–
Carmel, IN	459	3411.7	75,908	2	32	–	–
Gulfport, MS	460	3428.7	59,559	4	23	–	–
Waukesha, WI	461	3443.5	65,476	1	8	25	–
Rapid City, SD	462	3475.2	55,013	3	20	–	–
Oklahoma City, OK	463	3486.2	585,298	12	114	427	–
Bloomington, IL	464	3514.0	73,622	3	15	–	–
Perris, CA	465	3534.6	74,679	2	9	25	–
West Jordan, UT	466	3544.3	113,136	2	14	57	–
Jackson, MS	467	3545.0	132,215	7	39	–	–
Pharr, TX	468	3584.1	77,075	2	15	38	–
St. Joseph, MO	469	3615.3	61,275	2	14	38	–
Palm Bay, FL	470	3681.5	106,342	5	43	–	–
Edinburg, TX	471	3729.2	79,803	3	19	–	–
New Orleans, LA	472	3741.0	377,281	1	15	60	–
Midland, TX	473	3741.5	115,533	2	16	73	–
Lincoln, NE	474	3784.6	270,110	3	26	86	–
Tuscaloosa, AL	475	3862.5	84,424	1	21	–	–
Amarillo, TX	476	3900.6	182,518	2	25	73	–
Columbia, MO	477	3905.6	107,706	4	37	–	–
Huntsville, AL	478	3922.4	180,633	8	–	–	–
Brownsville, TX	479	3989.6	178,648	5	27	78	–
Wichita Falls, TX	480	4013.7	90,894	2	16	45	–
Odessa, TX	481	4087.1	102,381	2	11	36	–
Shreveport, LA	482	4100.1	163,611	9	61	–	–
Jacksonville, FL	483	4102.4	834,225	16	362	–	–
Port St. Lucie, FL	484	4118.5	192,631	10	–	–	–
Cape Coral, FL	485	4204.2	179,013	9	88	–	–
Surprise, AZ	486	4249.6	136,583	3	28	–	–
Virginia Beach, VA	487	4680.5	417,731	1	58	–	–
St. George, UT	488	4705.5	81,834	6	–	–	–
Austin, TX	489	4846.7	893,947	5	102	445	–
Tucson, AZ	490	4892.2	508,571	1	29	132	–
Waco, TX	491	4913.4	125,775	7	69	–	–
Palm Coast, FL	492	5050.1	77,271	11	–	–	–
Jacksonville, NC	493	5280.3	60,367	2	24	–	–
Duluth, MN	494	5486.4	66,602	3	12	26	–
Rio Rancho, NM	495	5950.8	92,607	6	36	–	–
Lawton, OK	496	6334.7	80,836	4	30	–	–
Abilene, TX	497	7097.8	109,596	5	30	–	–
Denton, TX	498	7297.7	121,649	3	22	–	–
Miramar, FL	499	11850.7	132,450	16	–	–	–
Anchorage, AK	500	34723.5	193,910	2	29	114	–

Note: '–' indicates no stores were added under that strategy for the corresponding city.
